# An Overture for eCAM: Science, Technology and Innovation Initiation for Prosperous, Healthy Nepal

**DOI:** 10.1093/ecam/nep156

**Published:** 2011-06-05

**Authors:** Krishna Kaphle, Dinesh Raj Bhuju, Pramod Kr Jha, Hom Nath Bhattarai

**Affiliations:** ^1^Department of Theriogenology, Institute of Agriculture and Animal Science, Rampur, Chitwan, Nepal; ^2^Nepal Academy of Science and Technology, Khumaltar, Lalitpur, Nepal

## Abstract

Nepal the “Shangri-La” in the lap of the Himalayas is gearing up for modern times as it starts rebuilding after a decade of senseless violence and destruction. The nation one of the poorest in the global development index is rich in natural resources and biodiversity. Reports of medicinal plants far exceeding those recorded and reported so far are encouraging and at the same time concerns for medicinal plants under threat as a result of overexploitation are emerging from Nepal. The harsh mountain terrains, lack of industrialization and harnessing potentiality of its areas of strength; water; natural resources and tourism make it poor in per capita income which averages ~ 300 US$, with half the population living under >1$ a day. Nepal is beginning to realize that the way ahead is only possible through the path of Science and Technology (ST). Nepal Academy of Science and Technology formerly known as Royal Academy of Science and Technology organized the fifth national conference held every 4 years that took place in the capital Kathmandu during November 10-12, 2008. The ST initiation event saw the participation of ~ 1400 people representing over 150 organizations from the country and experts from abroad. The theme for the fifth national meet was “Science, Technology and Innovation for Prosperous Nepal”. Complementary and Alternative Medicine (CAM) was an important theme in the event as the realization for the need of ST research focused in CAM for harnessing the chemo diversity potential was univocally approved.

Nepal the “Shangri-La” is nestled in the lap of the Himalayas rich in natural diversity. This small landlocked country is strategically situated between the two most populous nations in India and China and have land area of 147 181 km^2^, inhabited by nearly 30 million people. Topographically, Nepal is divided into three distinct ecological zones: mountains, hills and the plains. Nepal is rectangular in shape with the east–west length of 885 km, north-south width of 193 km and elevation ranging from 90 to 8848 m. The nation one of the poorest in the global development index is rich in natural resources and biodiversity. The harsh mountain terrains, lack of industrialization and harness of potentialities in its areas of strength; water; natural resources and tourism make it poor in per capita income which averages *∼*300 US$, with half the population living under >1$ a day. In the now common phrase “New Nepal making”, the claimed federal republic of Nepal in making is beginning to realize that the way ahead is only possible through the path of Science and Technology (ST). Nepal Academy of Science and Technology (NAST) formerly known as Royal Academy of Science and Technology in the initiation for adopting science organized the fifth national conference held every 4 years that took place in the capital Kathmandu during November 10–12, 2008. The event saw the participation of *∼*1400 people representing over 150 organizations from the country and experts from abroad. The theme for the fifth national meet was “Science, Technology and Innovation for Prosperous Nepal”.

The event saw the active participation of young researchers and a healthy proportion of females (25%), which is a good sign for the future of Nepalese ST. The technical section was spread over 80 sessions with 545 oral papers and 85 posters. Subject wise distribution of paper was agriculture and animal science (21%), biological science (14%), biotechnology and food science, health science, forestry and environment, chemical science, engineering and technology, information technology, physical and social sciences in respective strength.

One of the highlights of the meeting was the stress on the role of medicinal plants as potential strength for national development by the prime minister, finance minister and ST minister besides others. The other being the numbers of technical papers and posters related with the medicinal plants and traditional knowledge. Nepal as a true treasure house of diverse flora and fauna needs to invest in the research of the natural medicinal properties, document the traditional knowledge and strive for sustainable development adopting wise use of ST. Nepal's huge treasure of natural bioactive compounds and traditional knowledge on their use and application is a huge asset for the science of Complementary and Alternative Medicine (CAM) that needs to be studied and documented.

The research potentialities and technology transfer from abroad will be crucial in harnessing the huge treasure of medicinal plants, which the government of Nepal has placed in high priority [1]. Will Nepal be able to use its huge resource of natural resource of high-valued medicinal plants is to be seen in future, but there are ample examples from which it can learn as the world goes green to support its graying and growing population. The herbal medicine research initiative of Nepal is in its primitive stage and needs to learn from experience of countries in Asia like Taiwan [2], Korea and India [3] as we treasure our culture, traditional knowledge and yet pursue scientific basis of acceptance. Nepal surely presents itself as a natural laboratory for scientific research of CAM as it present unique materials worth detail investigation [4–6]. Medicinal herbs, traditional healing knowledge that has evolved in the fine blend of Traditional Chinese Medicine and Ayurveda yet with its own special touch to it, are unique for researchers in CAM science. Sustainable development is the way out for Nepal as it is now in the crossroad to jumpstart economic activities for growing population and at the same time sustains its fragile ecosystem. In the health-care system too, the need to highlight and adopt sustainable medicine for animals [7] and humans [8] is also urgent. The chaotic situation in the health-care system of Nepal demands that the traditional healing system and natural resources be judiciously evaluated and documented for the service of mankind [5]. In this NAST gathering, there were 49 oral and 11 posters papers on the related theme of phytomedicine. This shows the tremendous potentiality, interest and ongoing research in Nepal. With government's inclusion in one of the potential for growth, hopefully funding for research will see many works by Nepali scientists published and scientific evidences generated for CAM.

A paper on the theme “CAM and Scientific Evidences for Nepal's future” was presented by the editorial team from Evidence-Based Complementary and Alternative Medicine (eCAM) journal (K.K. and Edwin L. Cooper). This particular talk [1] set in the health research session, mentioned how scientific evidences are emerging for CAM and Nepal's unique position to contribute. It received huge inquiries and inputs on the existing CAM research activities in Nepal. The summarized abstract was as following:

The ancient healing art of complementary and alternative medicine (CAM) is now being subjected to rigorous repeated trials and replication of experiments in scientific lines. However, explanation of science is limited to the observations which again are an outcome of a time-space dimension of activities. Nepal's huge treasure of natural bioactive compounds and traditional knowledge on their use and application needs to be studied and documented. This will demand that technology is used to generate evidences that can explain and understand the science and logic behind it.

This brings us to the situation where the science of CAM, which is criticized for being woefully insufficient. Hence, it seems important to evaluate CAM and to find answers to the most pressing questions on how it works and concerns of its safety. There is no reason to say that it is unexplainable and measured in the glass of science as modern technology evolves. The science of herbal medicine has now received the impetus of the technological advancement where the bioactive compound isolation and its mechanism of action are beginning to be established. The philosophy of traditional medicine in the form of acupuncture, Yin-Yang, Meridians, Tridoshas, Chakras, Five-elements and many more are now beginning to be understood with their physiological basis. Terms like Bongham vessels, microvascular endothelial cells, cytokines (Th1/Th2) balance, cAMP/cGMP PDE activity besides the signal transduction elucidation of the cell are recent scientific basis of understanding CAM.

Hence, with technological advancement their development and use in CAM research as seen in countries like Korea, Japan, Taiwan, USA, China, India and others will enable Nepal to gear up for scientific evidence generation. The research potentialities and technology transfer from abroad will be crucial in harnessing the huge treasure of medicinal plants, which is priority in the government agenda. Nepal surely presents itself as a real nature's laboratory for the research of CAM and should also be a priority for scientific pursuit in a nation plagued by resource crunches. It is here where we can extend our collaborations with partner institutes from abroad to meet the global need for the growing, graying and greening population.

There are reports of medicinal plants in Nepal being more in numbers than those reported and at the same time some of them under the threat of overexploitation.

In this line, collaborative research on various themes of CAM is ongoing in Nepal. The increasing number of researchers with higher education degrees (PhDs) from various international universities will surely be vital in this aspect [9]. Bioprospecting research is one area where many of such researchers are engaged and sure enough it is in line with the demand of time [10]. It is for researchers abroad and elsewhere to approach NAST and set up collaborative links with individual researchers and institutions here in Nepal for evidence generation to support the science of CAM.

Works by Prof. Lindequist and her team with Nepali counterparts are worth mentioning here [6]. Their initial findings show the huge potentiality of antiviral-acting medicinal plants from Nepal. They researched on methanolic extracts of 41 plant species belonging to 27 families used in the traditional medicine in Nepal. Investigation was for *in vitro* antiviral activity against Herpes simplex virus type 1 (HSV-1) and influenza virus A by dye uptake assay in the systems HSV-1/Vero cells and influenza virus A/MDCK cells. The extracts of *Astilbe rivularis, Bergenia ciliata, Cassiope fastigiata* and *Thymus linearis* were reported to be potent anti-herpes viral activity. The extracts of *Allium oreoprasum, Androsace strigilosa, Asparagus filicinus, A. rivularis, B. ciliata* and *Verbascum thapsus* reportedly exhibited strong anti-influenza viral activity. Only the extracts of *A. rivularis* and *B. ciliata* demonstrated remarkable activity against both viruses'.

Steroidogenic (hormonal) roles are other areas of potent research from medicinal plants of Nepal [8]. Ongoing efforts with medicinal herbs have generated some evidence that needs to be scrutinized in rigorous scientific trials [11]. Likewise, Nepal should expose its cream scientists and researchers to the changes in evidence-based CAM research by bringing in international experts and arranging exchange visits. Not to mention, the promise of research funds have to be initiated at the earliest and competitive bids of proposals need to be invited. Setting up government initiative will help add value to its natural treasure that have huge potentiality as much needed revenue generating export item. However, the saying “nature's ability to provide for human need and not their greed” should be understood in sustainable harnessing of its valuable treasures [12] as we enter the era of New Nepal. ST is considered the tool for turning the struggling country around [13] and an overture for eCAM seems inevitable for uplifting the country.

## Challenges Ahead

The challenges facing this country is that it is ranked as one of the poorest and least developed country in the world with almost one-third of its population living below the poverty line (Ministry of Finance, 2006). Although it have considerable scope for exploring its potential in hydropower, eco-tourism and natural resources harnessing, prospects in trade and industry are hampered by its small economy. Nepal's technological backwardness, landlocked geographic location, remoteness, civil strife and susceptibility to natural disasters pose greatest challenges. Doubling of population density from 79 persons per square kilometer in 1971 to 157 persons per square kilometer in 2001 and an increasing population is another challenge for the small country [14]. The need to strengthen health system, including community-based case management of infections including neonates, establishment of referral linkage and provision of quality care at the referral level are some challenges in the health-care system. Private investment in health care is encouraging but their approach is not sustainable [15], likewise the medical research is just used as a con to avoid taxation. The recent outbreak of diarrhea in the remote areas of Nepal has exposed the disparity in the health-care facility and services provided by the government. High burden of infectious diseases illustrates the need to develop strategies for management of infections at the community level. Lack of basic sanitation and clean drinking water along with proper waste management strategies poses the greatest challenges in ensuring healthy Nepal.
